# Book reviews

**DOI:** 10.1155/S1463924691000159

**Published:** 1991


### Book reviews

### An Introduction to Laboratory Automation

By Victor Cerdá and Guillermo Ramis (John Wiley & Sons Ltd, Chichester, 1990). ISBN 0069-2883.

This is the second such book that has appeared recently attempting to cover the area of laboratory automation. Like its predecessor [1] it has been written by an academic group. Whilst it has many attributes, it suffers from this because it does not deal with the important aspects of economics, training and specification.

It concentrates on the nuts and bolts of the automation aspects of chemistry and provides good background reading material. However, the laboratory technician seeking to solve an automation problem is more likely to be put off by the detail given here than to use it to decide how best to automate. The book concentrates mainly on the electronic aspects of automation and could be extended to cover the detailed approach to automation problems. It is a helpful text, but has no references to examples, and is most suited for reading in an academic post-graduate environment.

### Reference

1. Valcarcel, M. and Luque de Castro, M. D., *Automatic Methods of Analysis, Techniques and Instrumentation in Analytical Chemistry*, Volume 9, Elsevier (1988).

**Peter B. Stockwell**

### A Guide to Materials Characterization and Chemical Analysis

Edited by John P. Sibilia (VCH Verlagsgesellschaft, Weinheim, Basel, Cambridge, New York, 1988). x + 318 pp. and 204 figures. DM 75.00, £25.95.

Written both for the novice and for the experienced scientist, this miniature encyclopaedia concisely describes over 100 materials methodologies, including evaluation, chemical analysis, and physical testing techniques. Each technique is presented in terms of its use, sample requirements, and the engineering principles behind its methodology. Real-life industrial and academic applications are also described to give the reader an understanding of the significance and utilization of the techniques of materials characterization. There is also a discussion of the limitation of each technique.

Highlights of the book are comprehensive coverage of polymer analysis, testing and evaluation, and descriptions of state-of-the-art technology in surface analysis and microscopy along with lucid illustrations and examples.

The book is of considerable assistance to the systems designer with guidelines on what best to use to construct a particular object. Produced in a handy size, the book will act as a good reference to solve problems as they arise.

